# Establishment of an innovative staging system for extramedullary plasmacytoma

**DOI:** 10.1186/s12885-016-2824-x

**Published:** 2016-10-08

**Authors:** Qian Zhu, Xiong Zou, Rui You, Rou Jiang, Meng-Xia Zhang, You-Ping Liu, Chao-Nan Qian, Hai-Qiang Mai, Ming-Huang Hong, Ling Guo, Ming-Yuan Chen

**Affiliations:** 1 Department of Nasopharyngeal Carcinoma, State Key Laboratory of Oncology in South China and Collaborative Innovation Center for Cancer Medicine, Sun Yat-Sen University Cancer Center, Guangzhou, Guangdong China; 2 Department of Nasopharyngeal Carcinoma, Sun Yat-Sen University Cancer Center, 651 Dongfeng Dong Road, Guangzhou, 510060 Guangdong China

**Keywords:** Extramedullary plasmacytoma, Clinical stage, Prognostic factors

## Abstract

**Background:**

Extramedullary plasmacytoma (EMP) is a rare malignant disease that lacks a unique clinical staging system to predict the survival of EMP patients and to design individualized treatment. Instead, clinicians have chosen to use the multiple myeloma (MM) staging system.

**Methods:**

Forty-eight EMP patients treated between 1996 and 2014 were included in this study. The new clinical stages were established according to independent survival factors using Cox regression model.

**Results:**

Lymph node metastasis and a larger primary tumor (≥5 cm) were the only two independent poor prognostic factors for overall survival (OS) and disease-free survival (*P* < 0.05). Stage I was defined as the disease without those two poor prognostic factors. Stage II was defined as the presence of either factor, and Stage III was defined as the presence of both factors. OS was significantly different in each stage of the new staging system (*P* < 0.001), with a median follow-up time for Stage I, Stage II and Stage III of 68, 23 and 14 months. The new staging system had enhanced prognostic value compared to the MM staging system (the area under ROC 0.763 versus 0.520, *P* = 0.044). Although no difference was observed between treatments in Stage I, the combination treatment was associated with a significantly beneficial OS in the late stages (5-year OS: 15.3 % versus 79.5 %; *P* = 0.032).

**Conclusions:**

The new staging system exhibited a promising prognostic value for survival and could aid clinicians in choosing the most suitable treatment for EMP patients.

## Background

Extramedullary plasmacytoma (EMP) is an extremely rare and discrete solitary mass of neoplastic monoclonal plasma cells, which was first described by Schridde in 1905 [1]. The incidence of EMP has been measured at 0.04 cases per 100,000 individuals [2] . Almost 80 % of EMPs are localized in the head and neck region [3, 4]. A previous study revealed that prognostic factors for EMP disease-free survival in the head-and-neck region were monoclonal immunoglobulin secretion and radiation administered to the CTV ≥45 Gy [5]. Tsang et al. [6] and Hollandet al. [7] suggested that EMP patients with tumors larger than 5 cm are at a higher risk of treatment failure. However, the independent prognostic factors for survival were unclear, making it impossible to establish a useful clinical staging system for EMP. Although clinicians use the international staging system (ISS) for multiple myeloma (MM), few reports have shown that the MM grading criteria can predict the prognosis of EMP patients. Additionally, the lack of uniform criteria for clinical staging made it difficult to predict the survival of EMP patients, design individualized treatment and compare the therapeutic efficacy between different countries and cancer centers.

The optimal management of EMP remains controversial. Radiotherapy plays an important role in the treatment of EMP [8]. Surgery can also be considered as an alternative first-line therapy [9]. However, radical excision is often difficult because of the size of the tumor and the proximity of vital organs. Furthermore, the role of chemotherapy in the treatment to reduce relapse rates or to improve survival rates remains unclear [10–12]. Generally, surgery and radiotherapy are effective treatments for EMP patients. However, clinicians still find it difficult to choose the optimal method for the management of EMP patients according to a unified standard.

The purpose of our study was to establish an innovative staging system according to a large consecutive cohort of patients with EMP who were diagnosed, treated and followed at the Sun Yat-Sen University Cancer Center.

## Methods

### Patients

Medical records of all patients treated for EMP at the Sun Yat-Sen University Cancer Center between 1996 and 2014 were retrospectively reviewed. For the use of human’s clinical data, prior patients’ consents and approval from Sun Yat-sen University Cancer Center Institutional Review Board were obtained. Patients were considered eligible for inclusion if they had a diagnosis of EMP based on a biopsy showing features characteristic of plasmacytoma, a negative skeletal survey, and a normal bone marrow biopsy. Patients with evidence of myeloma at the time of presentation were excluded. From those, 48consecutive patients were investigated. The diagnostic gold standard to diagnose the size of a metastasis lymph node and the primary tumor is imaging testing by Magnetic Resonance Imaging (MRI) or CT scanning. Positron Emission Tomography-Computed Tomography (PET-CT) was used to further identify suspicious lymph node metastases. Regional lymph node metastasis was diagnosed as the short radius equal to or more than 1 cm.

### Treatment

Treatment choices depended on the techniques available at the cancer center, the attending physician’s decision and the opinion of a multi disciplinary team (MDT). Patients in the study underwent single or combination treatments. The single treatments included surgery, radiotherapy, or chemotherapy alone, while combination treatments consisted of two or more treatment methods (surgery + radiotherapy, radiotherapy + chemotherapy, surgery + chemotherapy, surgery + radiotherapy + chemotherapy). In radical radiotherapy, gross tumor volume (GTV) was defined to encompass the entire tumor and regional metastatic lymph nodes. Clinical target volume (CTV) was defined to encompass the subclinical lesion around the entire tumor and regional metastatic lymph nodes. The surgical methods included endoscopic resection and open-approach resection. Depending on the myeloma guidelines, the chemotherapeutics included VAD (Vincristine + Adriamycin + Dexamethasone), MP (Melphalan + Prednisone) and MPT (Melphalan + Prednisone + Thalidomide). The CHOPP (Cyclophosphamide + Doxorubicin + Vincristine + Prednisone) adjuvant chemotherapy regimen was also included.

### Statistical analysis

All statistical analyses were performed using SPSS 16.0. The chi-squared test was used to investigate the relationship between lymph node metastasis and the clinicopathologic features of EMP. Overall survival was calculated by taking into consideration of all death events. Disease-free survival was calculated by considering only events that involved local recurrence, regional recurrence, distant metastasis or progressing to MM. Local relapse-free survival was calculated by considering only events of local recurrence at the primary site. Survival curves were plotted using the Kaplan-Meier method and compared using the log-rank test. To determine the independent prognostic factors for survival, the variables that reached *P* value <0.05 according to univariate analysis and potential influencing factor of survival (gender, age, number of primary tumor, treatment police and anatomic location of tumor) were subjected to Cox regression analyses. In all analyses, *P*-values < 0.05 were considered statistically significant.

### Establishment of EMP clinical stages and comparison with the multiple myeloma stage system

According to the multiple myeloma (MM) international staging system (ISS), stage I was defined as serumβ2-microglobulin less than 3.5 mg/L and serum albumin more than 35 g/L. Stage III was defined as serumβ2-microglobulingreaterthan 5.5 mg/L. Stage II was between stage I and stage III. An innovative EMP clinical stage was designed based on the combination of independent prognostic factors selected from the Cox model. Receiver operating characteristic (ROC) curves were used to compare the sensitivity and specificity of this new EMP clinical stage and the MM stage system for survival predictions.

## Results

### Patient clinicopathologic features

**Fig. 1:**
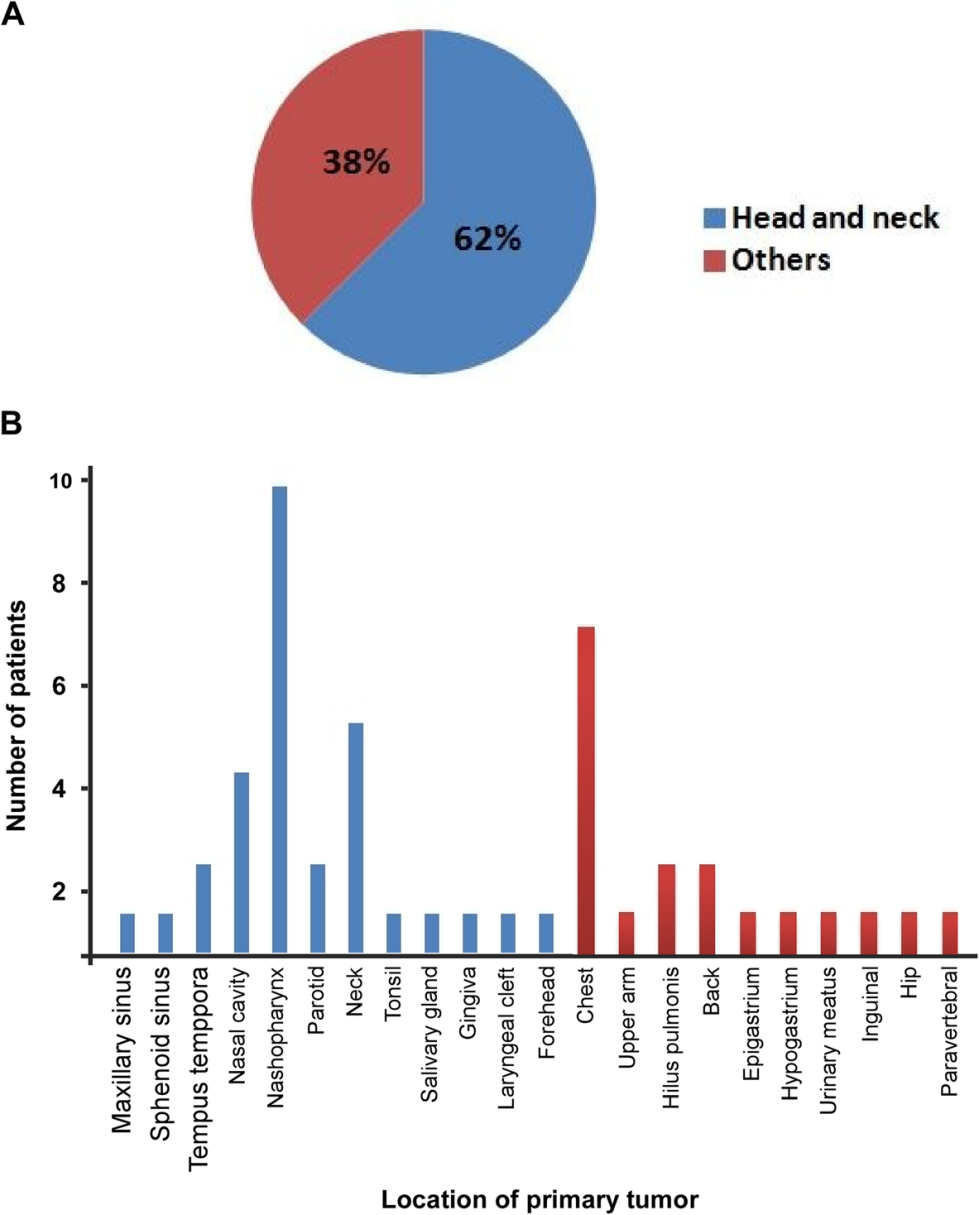
Anatomic location of the primary tumor in 48 extramedullary plasmacytoma patients. 48 EMP patients were included in this study. The initial location of 48 EMP patients were consisted of several sites, including 30(62 %) of head and neck and 18(38 %) of others

**Fig. 2:**
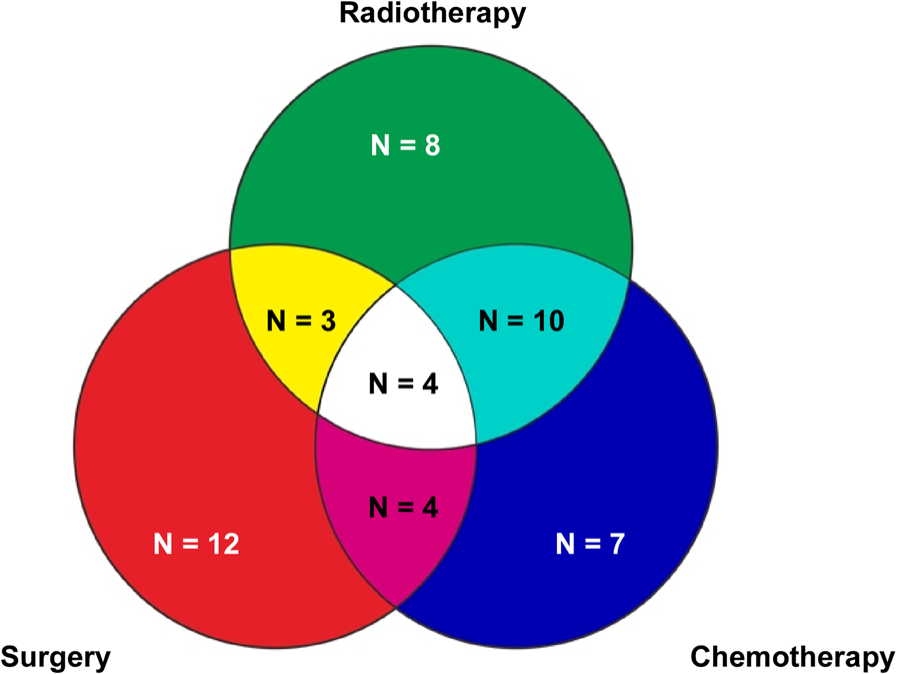
Details of treatments. Different colors represent different treatments. Among the 27 patients with single treatment, 8 patients received radiotherapy alone, 12 patients received surgery alone and 7 patients were treated with chemotherapy alone. Moreover, 21 patients underwent a combination treatment (*N* = number of patients)

The average age was 52 years, with a range of 20–75 years. The male-to-female ratio was 15:9. The initial location of EMP consisted of several sites, including 30(62 %) head and neck and 18(38 %) others (Fig. 1). Among those patients, 13(27 %) patients were diagnosed with lymph node metastasis. As shown in Table 1, there was no significant association between lymph node metastasis and patient’s age, gender, treatment, tumor location, tumor size or tumor number. Among the 27 patients with single treatment, 8 patients received radiotherapy alone, 12 patients received surgery alone and 7 patients were treated with chemotherapy alone. Moreover, 21 patients underwent a combination treatment. Although patients treated with radiotherapy were administered a dose of 1.8-2.2Gy per fraction, total doses ranged from 26 to 60Gy (median dose: 50Gy) for various tumor locations and different treatment policies. The treatment details are listed in Fig. 2.

**Table 1:** Association between lymph node metastasis and the clinicopathological features of EMP

Parameter	Number of patients (%)	Lymph node metastasis		*P*
		Yes	No	
Age, years				
<50	20 (41.7)	5 (25.0)	15 (75.0)	1.000
≥50	28 (58.3)	8 (28.6)	20 (71.4)	
Gender				
Male	30 (62.5)	9 (30.0)	21 (70.0)	0.740
Female	18 (37.5)	4 (22.2)	14 (77.8)	
Treatment				
Single	27 (56.2)	5 (18.5)	22 (81.5)	0.192
Combined	21 (43.8)	8 (38.1)	13 (61.9)	
Location				
Head and neck	30 (62.5)	10 (33.3)	20 (66.7)	0.317
Other	18 (37.5)	3 (16.7)	15 (83.3)	
Size of tumor				
<5 cm	24 (50.0)	6 (25.0)	18 (75.0)	1.000
≥5 cm	24 (50.0)	7 (29.2)	17 (70.8)	
Number of tumors				
Solitary	42 (87.5)	10 (23.8)	32 (76.2)	0.323
Sporadic	6 (542.8)	3 (50.0)	3 (50.0)

**Table 2:** Univariate analysis of patient characteristics for overall survival, disease free survival and local control among the 48 extramedullary plasmacytoma patients

Variable	Overall survival		Disease free survival		Local control	
	*P*	Regression coefficient (SE)	*P*	Regression coefficient (SE)	*P*	Regression coefficient (SE)
Gender (Male vs Female)	0.942	0.950 (0.695)	0.645	0.781 (0.535)	0.911	0.873 (1.225)
Age, years (<50 vs ≧ 50)	0.296	2.058 (0.691)	0.690	1.218 (0.495)	0.543	0.474 (1.228)
Lymph node (Without vs With)	0.005	11.767 (0.882)	0.014	5.438 (0.691)	0.483	2.698 (1.415)
Size of primary tumor (<5 cm vs ≧ 5 cm)	0.012	14.646 (1.071)	0.002	7.363 (0.646)	0.704	0.626 (1.232)
Number of primary tumors (Solitary vs Sporadic)	0.816	0.782 (1.056)	0.632	1.360 (0.641)	0.424	2.759 (1.270)
Treatment (Single vs Combined)	0.432	1.657 (0.643)	0.158	2.008 (0.494)	0.418	2.706 (1.230)
Anatomic location (HN vs Other)	0.095	2.981 (0.654)	0.485	1.413 (0.495)	0.979	1.033 (1.229)

*Abbreviation*: *HN* head and neck

### Overall survival

**Fig. 3:**
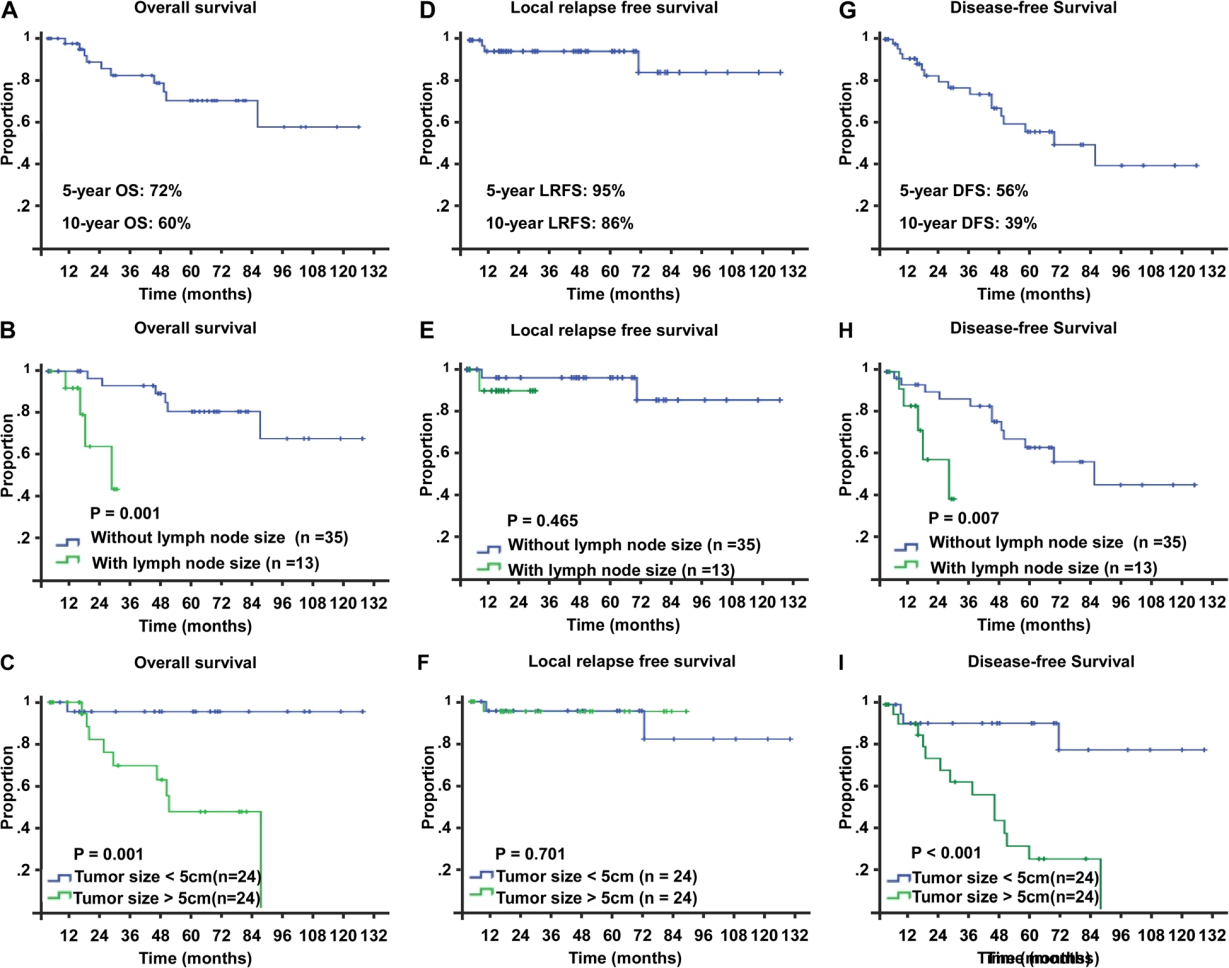
Survival curves in 48 Extramedullary Plasmacytoma Patients. Overall survival (**a**), local relapse free survival (**d**) and disease-free survival (**g**) for 48 EMP patients and overall survival (**b**), local relapse free survival (**e**) and disease-free survival (**h**) according to the patients with lymph node metastasis (*n* = 13) or without lymph node metastasis (*n* = 25). Overall survival (**c**), local relapse free survival (**f**) and disease-free survival (**i**) between EMP patients with a tumor equal to or more than 5 cm (*n* = 24) or less than 5 cm (*n* = 24)

The median follow-up time for patients was 44.5 months. The overall 5-year and 10-year survival rates were 72 % and 60 %, respectively (Fig. 3a). At the last follow-up, 10 patients had died. Among those patients, 8 (80 %) had died of the disease, whereas 2 patients (20 %) died of other causes. EMP patients with lymph node metastasis were associated with a significantly poorer overall survival (OS) compared with those without lymph node metastasis (median survival time: 14.0 *versus* 60.0 months, respectively, *P* = 0.001; Fig. 3b). Moreover, EMP patients with a primary tumor ≥5 cm had a significantly poorer overall survival (OS) compared with tumor sizes less than 5 cm (median survival time: 25.0 versus 53.5 months, *P* = 0.001; Fig. 3c). Additionally, lymph node metastasis and the size of the primary tumor were independent prognostic factors for poorer OS in EMP patients (*P* = 0.019 and *P* = 0.026, respectively). However, the number of primary tumors, the anatomic location of the primary tumor and the choice of treatment were not significantly associated with the OS of EMP patients (Tables 2 and 3). Subgroup analysis of 25 patients treated with radiotherapy showed patients treated with total dose greater than 45Gy had higher OS than the patients treated with total dose less than or equal to 45 Gy (median survival time: 53.5 versus 23.0 months, respectively, *P* = 0.017).

**Table 3:** Prognostic factors of overall survival, disease free survival and local control among the 48 extramedullary plasmacytoma patients

Variable	Overall survival			Disease free survival		
	*P*	Relative risk	95 % CI	*P*	Relative risk	95 % CI
I						
Gender (Male vs Female)	0.382	2.287	0.358–14.613	0.940	0.953	0.276–3.297
Age, years (<50 vs ≧ 50)	0.375	2.282	0.369–14.112	0.752	1.216	0.362–4.085
Lymph node metastasis (Without vs With)	0.019	10.335	1.463–72.986	0.048	4.279	1.014–18.052
Size of primary tumor (<5 cm vs ≧5 cm)	0.026	18.612	1.416–244.713	0.004	7.496	1.903–29.534
Number of primary tumors (Solitary vs Sporadic)	0.480	2.223	0.242–20.455	0.723	0.770	0.182–5.074
Treatment (Single vs Combined)	0.650	0.728	0.185–2.872	0.910	0.940	0.321–2.754
Anatomic Location (HN vs Others)	0.306	2.096	0.508–8.647	0.988	1.008	0.356–2.856
II						
Gender (Male vs Female)	0.378	2.276	0.364–14.230	0.990	1.008	0. 300–3.388
Age, years (<50 vs ≧ 50)	0.411	2. 049	0.371–11.311	0.807	1.159	0.354–3.799
Clinical stage (I vs II vs III)	0.001	13.030	2.812–60.382	<0.001	5. 795	2.245–14.958
Number of primary tumors (Solitary vs Sporadic)	0.472	2.248	0.247–20.461	0.746	0.791	0.191–3.272
Treatment (Single vs Combined)	0.680	0.680	0.190–2.957	0.982	0.988	0.342–2.851
Anatomic location (HN vs Others)	0.291	2.291	0.521–8.842	0.951	1.033	0.365–2.924

*Abbreviation*: *HN* head and neck

### Local relapse-free survival

Local recurrences developed in 6.3 % of patients (3 of 48). The overall 5- and 10-year local relapse-free survival (LRFS) rates were 95 % and 86 %, respectively (Fig. 3d). In the 3 patients who experienced local recurrence, 2 patients who underwent radiotherapy and adjuvant chemotherapy relapsed within 7 months, while the patient treated with surgery alone relapsed 5 years later. However, there was no significant association between the LRFS and the choice of treatment (*P* = 0.399). Tumor size and lymph node metastasis were not correlated with the LRFS of EMP patients (*P* = 0.465 and *P* = 0.701, Fig. 3e and f, respectively). The initial site of the tumor, age, gender, treatment of EMP patients, tumor number and metastasis lymph node number were also not prognostic factors for the LRFS of EMP patients. Subgroup analysis showed there was no association between total radiation dose and LRFS (*P* = 0.885).

### Disease-free survival

The 5-year and 10-year DFS rates were 56 % and 39 %, respectively (Fig. 3g). Two patients progressed into MM within 5 years of the initial diagnosis. Our analysis revealed significant associations between lymph node metastasis and poorer DFS in EMP patients (median follow-up time: 14 months versus 49 months, respectively, *P* = 0.007; Fig. 3h). The group with a primary tumor equal or more than 5 cm in size had poorer DFS rates than in the group with tumors less than 5 cm (*P* < 0.001; Fig. 3i). Further analysis showed that lymph node metastasis and primary tumor size were significant prognostic factors for DFS (*P* = 0.048 and *P* = 0.004, respectively), whereas age, gender, initial location of the tumor and treatment were not predictive. In the subgroup analysis of patients treated with radiotherapy, patients treated with total dose > 45Gy had higher DFS than the patients treated with total dose ≦ 45 Gy (median follow-up time: 47.5 versus 18.0 months), while *P*-value was not detected (*P* = 0.267).

### Establishment of innovative EMP staging systems and comparison to the MM staging

**Fig. 4:**
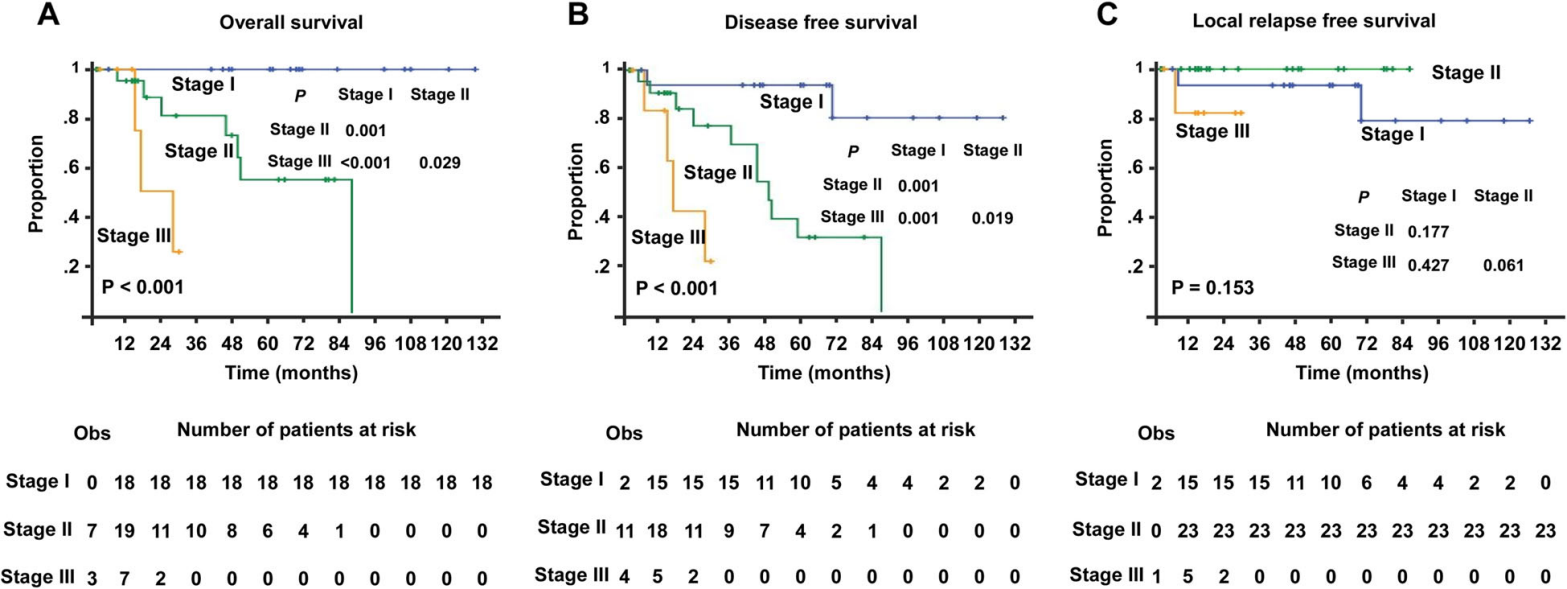
Comparison of survival according to the new clinical staging system in Extramedullary Plasmacytoma patients. Present study analyzed the overall survival (**a**), disease-free survival (**b**) and local control (**c**) between different clinical stages. The small vertical tick marks of “Obs” represented the observed number of events patients. “Number of patients at risk” represented number of patient possible happened events in the follow-up time

According to the independent survival factors of EMP patients and the similar HR of primary tumor size and lymph node metastasis (Table 3), an innovative clinical staging for EMP was classified into three grades. Stage I was defined as a primary tumor size less than 5 cm without lymph node metastasis. Stage II was defined as primary tumor size less than 5 cm with lymph node metastasis or a primary tumor equal to or larger than 5 cm without lymph node metastasis. Stage III was defined as primary tumor size equal to or larger than 5 cm combined with lymph node metastasis. Using this new EMP clinical staging system, 18, 23, and 7 patients were staged to Stage I, II, and III. All patients in Stage I was still alive at the last follow-up. Seven of 23 (30.4 %) patients in Stage II died. The mortality of Stage III was 42.9 % (3/7). Further analysis revealed patients clinical stage was significantly associated with overall and disease-free survival (both *P* < 0.001; Fig. 4a, b). The median follow-up time of patients diagnosed as Stage I, Stage II and Stage III were 68, 23 and 14 months, respectively. Moreover, statistical significant difference of overall survival was detected between Stage I and Stage II (*P* = 0.001), Stage I and Stage III (*P* < 0.001), Stage II and Stage III (*P* = 0.029) (Fig. 4a). Similarly, statistical significant difference of disease-free survival was found between Stage I and Stage II (*P* = 0.001), Stage I and Stage III (*P* = 0.001), Stage II and Stage III (*P* = 0.019) (Fig. 4b). Additionally, clinical stage could also be an independent factor for poorer OS and DFS (*P* = 0.001 and *P* < 0.001, Table 3).

**Fig. 5:**
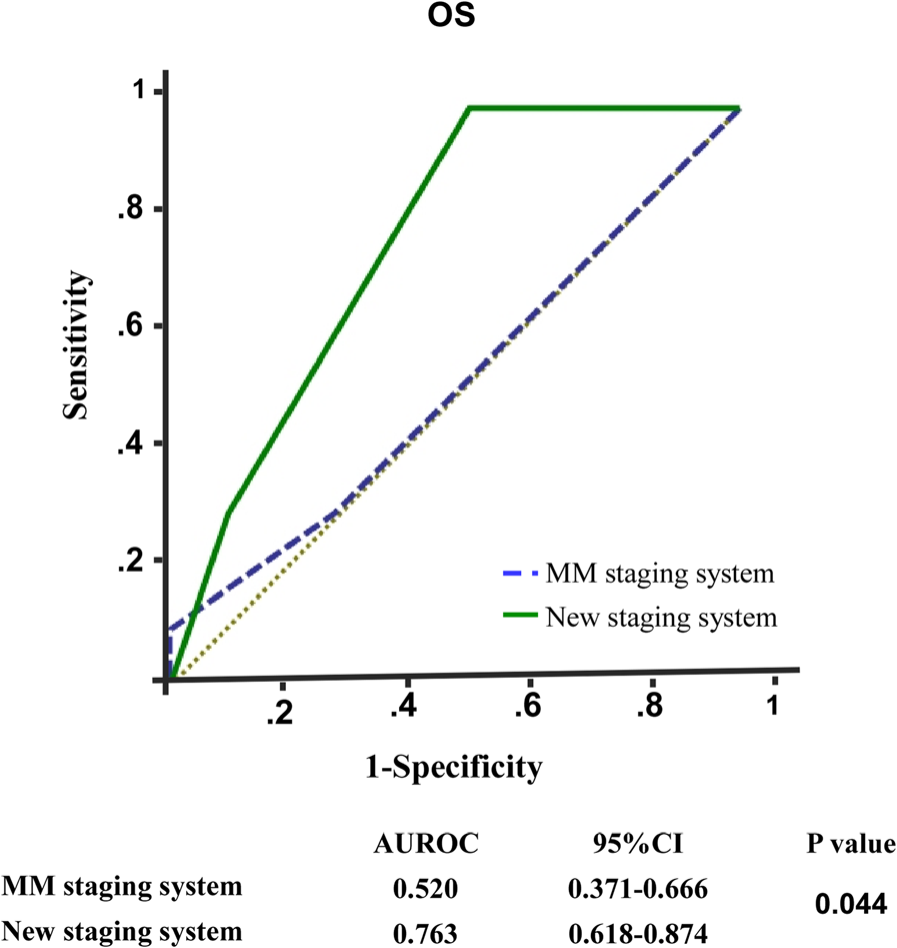
Comparisons of the sensitivity and specificity for the prediction of overall survival. The area under the receiver operating characteristic (AUROC) curves was used to compare the sensitivity and specificity for the prediction of overall survival between the multiple myeloma staging system and new staging model

However, 34 and 13 patients were staged in Stages I or II based on the MM clinical staging system, respectively. The median follow-up time of patients with Stage I and Stage II were 44 and 40 months, respectively. Only one patient was diagnosed in Stage III, who died after 7 years of disease progression and treatment failure. However, there were no significantly associations between MM stage and OS/DFS (*P* = 0.744 and *P* = 0.815, respectively). Moreover, ROC analysis showed that the present EMP staging system exhibited a better prognostic value for OS than the MM staging system. The areas under the curves were 0.763 versus 0.520, *P* = 0.044 (Fig. 5).

### The clinical significance of EMP clinical stage

**Fig. 6:**
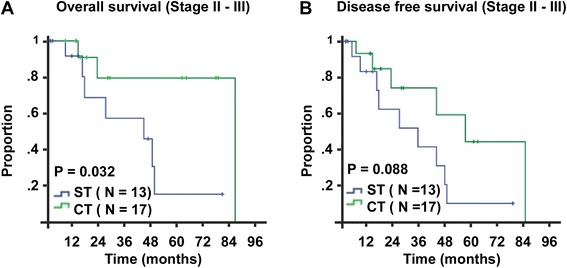
Survival curves of different treatments in EMP patients with late stage. According to the new staging model, 30 EMP patients were classified into late stage (Stage II–III). Patient in this stage treated with different treatment had different overall survival and disease free survival. “ST” represented single treatment and “CT” represented combined treatment

Subgroup analysis of the late stages (Stages II and III) showed that the patients treated with single therapy had poorer OS and DFS than the patients treated with combined therapy (5-year OS: 15.3 % versus 79.5 %, *P* = 0.032; 5-year DFS: 10.4 % versus 44.5 %, *P* = 0.088, respectively, Fig. 6a and b). However, in stage I, 13 patients treated with a single treatment and 5 treated with the combined treatment survived until the last follow-up time.

## Discussion

EMP is an extremely rare malignant disease. The lack of a unified staging criteria system makes it difficult to predict survival outcome and to define treatment choice. The present study analyzed a large cohort (48 patients) with a long follow-up, allowing us to draw reliable conclusions with regard to prognostic factors in EMP. The OS rates for 5-year (72 %) and 10-year (60 %), and the 5-year (56 %) and 10-year DFS(39 %) were similar to that of other series [12–14]. Therefore, the results from our population are comparable to those previously described. This study showed that large primary tumor and lymph node metastasis were independent prognostic factors for survival. According to the prognosis factors and similar relative risks, the EMP patients were classified into three grades. This staging system had a better prognostic value for OS than the MM staging system. Furthermore, this new staging system can select high-risk EMP patients and help design individualized therapeutic regimens.

Although EMP can arise throughout the body, almost 90 % of tumors arise in the head and neck, especially in the upper respiratory tract [9, 13–16]. The rate of cervical lymph node involvement for patients with EMP of the head and neck varies between 10 % and 15 % [17]. In a previous report, the presence of a cervical lymph node plasmacytoma should suggest an upper respiratory tract or oropharynx plasmacytoma rather than a primary lymph node plasmacytoma [18]. This study showed that the presence of lymph node metastasis was indicative of a primary tumor, although the size and location of the primary tumor were different. Furthermore, patients with lymph node metastasis had a shorter survival time compared to those without lymph node metastasis. Additionally, lymph node metastasis was an independent prognostic factor for EMP patients. Based on these observations, lymph node metastasis was the first factor included in our staging system. Ryohei et al. confirmed that tumor size was not a significant factor for local control in 42 EMP patients [19]. Tsang et al. [6] and Holland et al. [7] suggested that patients with tumors more than 5 cm are at higher risk of treatment failure. In the present study, patients with a tumor equal to or more than 5 cm had shorter OS and DFS. Moreover, tumor size may be an independent prognostic factor for poorer OS and DFS in patients with EMP. Based on these results, tumor size was the second factor considered in our staging system.

Some authors believe that EMP and MM are different phases of the same disease process [20] and used the same clinical grading criteria, whereas others believe that they are different diseases. If solitary EMP is an initial stage of MM, chemotherapy might play a more important role in management of the disease [21]. However, several studies showed that chemotherapy does not reduce relapse rates or improve survival rates and, at present, has no role in the primary management of EMP [10–12]. Moreover, in this study, only 2 (2/48) patients progressed to MM within 5 years. This fact prompted us to develop the specialized staging system for EMP. As shown in our study, the survival curves were distinctly different between the clinical stages. The staging system is a significant independent prognostic factor for OS. Furthermore, the comparison of the new staging system and the MM staging system showed a better prognostic value for OS.

Radiotherapy is a basic/primary treatment for EMP [2]. One study showed that a dose greater than 45Gy to the target volume improves the local control of EMP in the head and neck [19]. In our study, patients treated with total dose greater than 45Gy were showed higher OS than the patients treated with total dose less than or equal to 45 Gy. However, there were no association between total dose and DFS/LRFS, which may be influence by the diversity of combination treatment polices. Surgery can also achieve a high rate of local control in certain situations [9]. In our study, 41 (41/48) patients were treated with radiotherapy or surgery, and the overall 5- and 10-year LRFS rates were 95 % and 86 %, respectively. This result confirmed that radiotherapy and surgery play critical roles in the treatment of EMP. However, the surgical margin of EMP still lacks unified standards, which need further study. The UK Myeloma Forum has suggested that adjuvant chemotherapy is considered for EMP in the following cases: patients with tumors larger than 5 cm, patients with high-grade tumors, patients with refractory and/or relapsed disease, and patients with MM [7]. The present analysis found that patients treated with the simple treatment regimen had poorer OS and DFS than the patients treated with the combined treatment in the late stages (Stage II and III). Using this novel clinical staging, we can identify high-risk patients, which may help to design more aggressive therapeutic regimens and improve the overall survival rate in EMP patients. However, we could not put forward the exact combination treatment scheme for the limited number of patients in subgroup analysis.

This retrospective study and the method of determining the criteria for the stages had several limitations. First, this study demonstrated independent survival factors for EMP patients involving long time spans and a heterogeneous radiotherapy technique. Second, for the limited number of patients in the subgroup, further prospective or larger numbers of cases are required.

## Conclusions

In this study, we found that a large primary tumor (≥5 cm) in combination with lymph node metastasis were independent poor prognostic factors for OS and DFS in EMP patients. The innovative EMP clinical staging based on those two factors exhibited better prognostic value for EMP patient survival than the MM staging system and could aid clinicians in choosing the most suitable treatment. Based on the current findings it may be worth to consider the innovative EMP clinical staging system.

## Abbreviations

CTV: Clinical target volume
DFS: Disease-free survival
EMP: Extramedullary plasmacytoma
GTV: Gross tumor volume
ISS: International staging system
LRFS: Local relapse-free survival
MM: Multiple myeloma
OS: Overall survival
